# Comparison of Seven-Day Versus Continuous Prophylactic Antibiotic Therapy Until Delivery in Preterm Premature Rupture of Membranes

**DOI:** 10.7759/cureus.83991

**Published:** 2025-05-12

**Authors:** Guddad Shabana Hameed, Shobha Shirgur, Mallanagouda Patil, Rajasri G Yaliwal, Neelamma Patil

**Affiliations:** 1 Obstetrics and Gynecology, Shri BM Patil Medical College Hospital and Research Centre, Vijayapura, IND; 2 Pediatrics, Shri BM Patil Medical College Hospital and Research Centre, Vijayapura, IND

**Keywords:** antibiotics, maternal outcomes, neonatal outcomes, pprom, prophylactic antibiotics

## Abstract

Background and aim

Preterm prelabour rupture of membranes (PPROM) refers to the spontaneous rupture of fetal membranes before the onset of labor and prior to 37 completed weeks of gestation. PPROM is associated with significant maternal and neonatal complications. Maternal risks include chorioamnionitis, abruptio placentae, and postpartum infections. Neonatal complications commonly observed are respiratory distress syndrome (RDS), neonatal sepsis, cerebral palsy, and necrotizing enterocolitis (NEC). This study aimed to evaluate and compare maternal and neonatal outcomes in women with PPROM treated with prophylactic antibiotics for seven days versus antibiotics administered until delivery.

Materials and methods

This comparative study included 110 pregnant women between 26 weeks 0 days and 36 weeks six days of gestation. Participants were divided into the following two groups: group 1 received prophylactic antibiotics for seven days, and group 2 received antibiotics until delivery. Data collected included the duration of membrane rupture, types of antibiotics used, and various maternal and neonatal outcomes.

Results

A significantly lower incidence of persistent amniotic fluid leakage was observed in group 1 (31; 56.4%) compared to group 2 (45; 81.8%) (p<0.002). Continuous positive airway pressure (CPAP) support was not required in 41 (74.5%) of neonates in group 1 and 40 (72.7%) in group 2. However, a significantly higher proportion of neonates in group 2 required high-flow nasal cannula (HFNC) support compared to group 1 (p=0.015). Additionally, a shorter neonatal hospital stay (one to three days) was more frequent in group 1 (29; 52.7%) than in group 2 (17; 30.9%) (p=0.048).

Conclusion

A seven-day course of prophylactic antibiotics in PPROM is as effective as continuous antibiotic therapy until delivery, with added benefits of reduced neonatal hospital stay and potentially fewer antibiotic-associated risks.

## Introduction

Preterm prelabour rupture of membranes (PPROM) is defined as the spontaneous rupture of fetal membranes before the onset of labor and prior to 37 completed weeks of gestation [1]. It complicates approximately 2-3% of all pregnancies and is responsible for a significant proportion of preterm births, contributing to increased maternal and neonatal morbidity and mortality [2]. The pathophysiology of PPROM is complex and multifactorial. It is often associated with intrauterine infection, oxidative stress-induced DNA damage, premature cellular senescence, and inflammatory processes within the fetal membranes. Additional risk factors include poor prenatal care, nutritional deficiencies, low maternal body mass index, low socioeconomic status, sexually transmitted infections, vaginal bleeding, and tobacco use [3,4].

PPROM is not only a leading cause of preterm delivery but also carries serious clinical implications for both mothers and neonates [5]. Maternal complications include chorioamnionitis, placental abruption, and postpartum endometritis [2]. Neonates born following PPROM face an elevated risk of adverse outcomes such as respiratory distress syndrome (RDS), neonatal sepsis, cerebral palsy, and necrotizing enterocolitis (NEC) [6].

The use of prophylactic antibiotic therapy in women with PPROM has been shown to prolong the latency period (the time between membrane rupture and delivery), reduce ascending infections, and improve neonatal outcomes [7,8]. Antibiotic administration is particularly important in preventing microbial invasion of the amniotic cavity, thus decreasing the risk of both maternal and neonatal sepsis. While several broad-spectrum antibiotic regimens have demonstrated efficacy in this context, there is no clear consensus on the optimal duration of therapy [9,10].

Clinical practices vary widely; some protocols advocate for a standard seven-day course of antibiotics, while others recommend continuing antibiotic therapy until delivery. Emerging evidence suggests that prolonged antibiotic use may further reduce infectious complications and prolong pregnancy latency, but this approach must be balanced against potential risks such as antibiotic resistance or alteration of maternal and neonatal microbiota [9]. Therefore, evaluating the efficacy and safety of different durations of antibiotic treatment remains a key clinical question in the management of PPROM.

In light of these considerations, the present study aimed to compare maternal and neonatal outcomes in women with PPROM who were treated with a combination of intravenous ceftriaxone, intravenous metronidazole, and oral clarithromycin, using two antibiotic protocols - one with a fixed duration of seven days and the other continued until delivery - in order to determine the more beneficial approach for improving perinatal outcomes.

## Materials and methods

This prospective randomized controlled study was conducted from August 2024 to April 2025 at Shri BM Patil Medical College Hospital and Research Centre, Bijapur Lingayat District Educational (BLDE) (Deemed to be University), Vijayapura, India, after obtaining ethical approval from the Institutional Ethics Committee (BLDE/IEC/893/2022-23) and was registered with the Clinical Trials Registry, India (CTRI/2024/08/072969). A total of 110 pregnant women diagnosed with preterm prelabour rupture of membranes (PPROM) between 26 weeks 0 days and 36 weeks six days of gestation were enrolled after providing written informed consent. Participants were randomly allocated into two groups of 55 each using simple random sampling. Group 1 received a fixed seven-day course of prophylactic antibiotics, while group 2 received antibiotics until delivery. The primary aim was to compare maternal and neonatal outcomes between the two regimens, particularly focusing on latency period, infection rates, perinatal complications, and symptoms of chorioamnionitis. All participants were regularly monitored for clinical signs of chorioamnionitis, including fever, uterine tenderness, and maternal tachycardia, which were documented at the time of admission and during the course of the study.

Inclusion criteria were pregnant women aged 18 years or older with singleton pregnancies and a gestational age between 26 weeks 0 days and 36 weeks six days, confirmed by early ultrasound or a reliable last menstrual period. Eligible participants had a clinical diagnosis of PPROM made within 72 hours prior to enrolment, with cervical dilatation less than 3 cm at the time of presentation. Exclusion criteria included pregnancies complicated by fetal anomalies, abnormal placentation, or maternal comorbidities such as diabetes mellitus or hypertensive disorders of pregnancy. Women with a history of cervical incompetence, previous or current cervical cerclage, or documented urogenital tract infections at the time of admission were also excluded from the study.

The antibiotic regimen was standardized in both groups. Group 1 received intravenous ceftriaxone 1 g every 12 h and intravenous metronidazole (Metrogyl) three times daily for three days, followed by oral clarithromycin 500 mg twice daily for a total of five days. In group 2, the same initial regimen was administered, but oral clarithromycin was continued twice daily until delivery. The choice of these antibiotics was based on broad-spectrum coverage targeting common vaginal flora and pathogens associated with intra-amniotic infection. All women received standard obstetric care and monitoring per hospital protocol during their admission.

**Figure 1:**
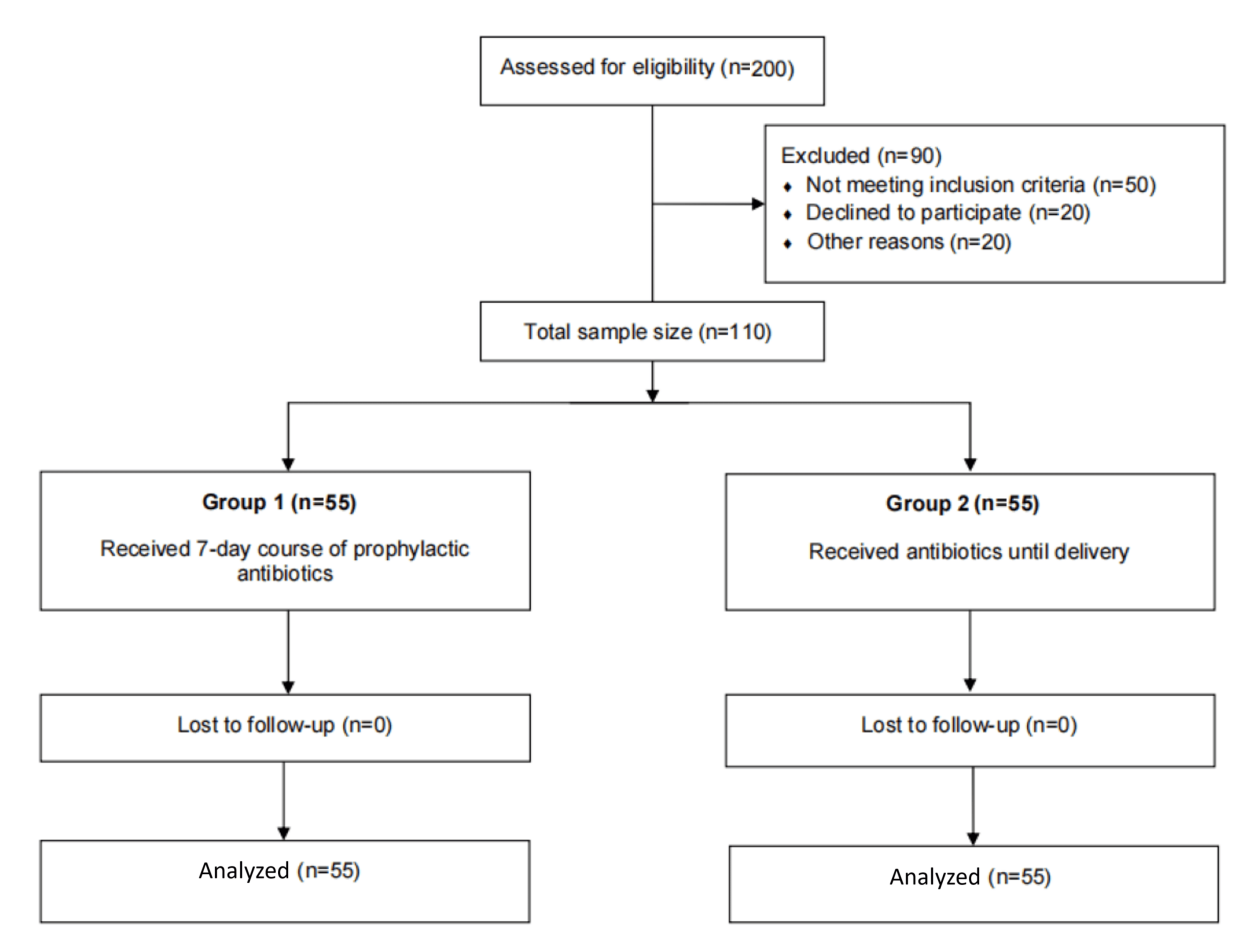
Flowchart of study participant enrollment, exclusion, follow-up, and analysis.

Sample size was calculated using G*Power software version 3.1.9.7 (Düsseldorf, Germany: Heinrich Heine University Düsseldorf), based on previously reported differences in neonatal outcomes (7.7% in seven-day treatment versus 3.1% in extended treatment). To achieve 95% power with a two-tailed alpha of 0.05, a minimum of 110 participants (55 per group) was required. Data were collected using Microsoft Excel (Microsoft Corp.: Redmond, WA) and analyzed using SPSS version 20 (IBM Corp.: Armonk, NY). Categorical variables were presented as frequency and percentage and compared using the chi-square test. A p-value of less than 0.05 was considered statistically significant (Figure 1).

## Results

This study included 110 pregnant women diagnosed with preterm prelabour rupture of membranes (PPROM), equally divided into the following two groups: group 1 received prophylactic antibiotics for seven days, while group 2 continued antibiotics until delivery.

A majority of participants in both groups were in the 21-25 years age category, with no significant difference in age distribution between the groups. Most women were multigravida, accounting for 72.7% in the seven-day antibiotic group and 63.6% in the until-delivery group. Gestational age at admission was slightly higher in the until-delivery group, with over two-thirds presenting between 32 and 36 weeks, though this difference was not statistically significant (Table 1).

**Table 1:** Sociodemographic characteristics of study participants. Categorical variables were presented as frequency and percentage and compared using the chi-square test. A p-value of less than 0.05 was considered statistically significant.

Variable	Category	Group 1 (antibiotics for 7 days) (n=55)	Group 2 (until delivery) (n=55)	Chi-square value	p-Value
Age	<20 years	8 (14.5%)	10 (18.2%)	2.0642	0.5592
	21-25 years	29 (52.7%)	24 (43.6%)		
	26-30 years	14 (25.5%)	13 (23.6%)		
	>30 years	4 (7.3%)	8 (14.6%)		
Obstetric score	Primigravida	15 (27.3%)	20 (36.4%)	1.0476	0.3061
	Multigravida	40 (72.7%)	35 (63.6%)		
Gestational age	24-28 weeks	7 (12.7%)	3 (5.4%)	3.0402	0.2186
	28-32 weeks	19 (34.5%)	15 (27.3%)		
	32-36 weeks	29 (52.7%)	37 (67.3%)		

Cervical dilation at admission also differed notably between groups; 76.3% of the seven-day group had a closed cervix compared to only 20.0% in the until-delivery group. Mode of delivery varied significantly, with more vaginal births in the seven-day group (65.5% versus 34.5%) and more cesarean deliveries in the until-delivery group (65.5% versus 34.5%). Notably, no maternal complications were observed in either group during the study period (Table 2).

**Table 2:** Maternal clinical characteristics. Categorical variables were presented as frequency and percentage and compared using the chi-square test. A p-value of less than 0.05 was considered statistically significant. LSCS: lower segment cesarean section

Variable	Category	Group 1 (antibiotics for 7 days) (n=55)	Group 2 (until delivery) (n=55)	Chi-square value	p-Value
Amniotic fluid	Clear	55 (100.0%)	54 (98.1%)	1.0091	0.3151
	Meconium stained	0 (0%)	1 (1.9%)		
Amniotic fluid index	Nil	0	3 (5.4%)	15.76	<0.003
	<5 cm	4 (7.3%)	8 (14.6%)		
	6-10 cm	18 (32.7%)	9 (16.4%)		
	>10 cm	11 (20%)	2 (3.6%)		
	Normal	22 (40%)	33 (60%)		
Cervical dilation	0 cm	42 (76.3%)	11 (20.0%)	41.8945	<0.0001
	1 cm	12 (21.8%)	19 (34.6%)		
	2 cm	1 (1.9%)	21 (38.1%)		
	3 cm	0 (0%)	4 (7.3%)		
Mode of delivery	Vaginal	36 (65.5%)	19 (34.5%)	10.5091	0.0012
	LSCS	19 (34.5%)	36 (65.5%)		

Rates of neonatal intensive care unit (NICU) admission were comparable across both groups, with 47.3% in the seven-day group and 41.8% in the until-delivery group. Among admitted neonates, the most common indication was low birth weight combined with respiratory distress syndrome (RDS), especially in the seven-day group (73.1%). However, differences in indication patterns were not statistically significant (Table 3).

**Table 3:** Comparison of NICU admission between study groups. Categorical variables were presented as frequency and percentage and compared using the chi-square test. A p-value of less than 0.05 was considered statistically significant. NICU: neonatal intensive care unit; LBW: low birth weight; RDS: respiratory distress syndrome

Variable	Category	Group 1 (antibiotics for 7 days) (n=55)	Group 2 (until delivery) (n=55)	Chi-square value	p-Value
NICU admission	Yes	26 (47.3%)	23 (41.8%)	0.3312	0.5649
	No	29 (52.7%)	32 (58.2%)		
Reason for admission	Preterm + RDS	7 (26.9%)	10 (43.5%)	0.6261	0.4287
	LBW + RDS	19 (73.1%)	13 (56.5%)	1.5865	0.2078

Respiratory support patterns showed some variation. The requirement for high-flow nasal cannula (HFNC) support differed significantly between groups, with fewer neonates in the until-delivery group requiring one to two days of HFNC (11.0% versus 29.0%, p=0.04). CPAP and oxygen hood usage did not differ significantly, though oxygen hood was used slightly more frequently and for longer durations in the until-delivery group (Table 4).

**Table 4:** Neonatal respiratory support. Categorical variables were presented as frequency and percentage and compared using the chi-square test. A p-value of less than 0.05 was considered statistically significant. CPAP: continuous positive airway pressure; HFNC: high-flow nasal cannula; O_2_: oxygen

Variable	Category	Seven days	Until delivery	Chi-square value	p-Value
CPAP use	0 days	41 (74.5%)	40 (72.7%)	2.0790	0.5562
	1-2 days	8 (14.5%)	7 (12.7%)		
	3-5 days	6 (11.0%)	6 (11.0%)		
	>5 days	0 (0%)	2 (3.6%)		
HFNC	0 days	37 (67.3%)	44 (80.0%)	6.4361	0.04
	1-2 days	16 (29.0%)	6 (11.0%)		
	3-5 days	2 (3.7%)	5 (9.0%)		
O₂ hood	0 days	40 (72.7%)	46 (83.6%)	4.6408	0.0982
	1-2 days	13 (23.6%)	5 (9.0%)		
	>3 days	2 (3.7%)	4 (7.4%)		

Neonatal outcomes, in terms of early rooming-in and survival, favored the until-delivery group, though not significantly. Four neonatal deaths were recorded in the seven-day group, while none occurred in the until-delivery group. The causes of neonatal death in group 1 were extremely low birth weight <1 kg (n=4). Hospital stay duration (from birth to discharge) varied significantly, with a higher proportion of neonates in the until-delivery group requiring prolonged stays (>4 days) compared to the seven-day group (p=0.0202). Neonates with short interval from PPROM to delivery (less than 24 hours) had more favorable outcomes, including lower rates of respiratory support and shorter hospital stays, while prolonged interval from PPROM to delivery (more than 48 hours) was associated with higher respiratory support needs and longer hospital stays (Table 5).

**Table 5:** Neonatal final outcome and hospital stay. Categorical variables were presented as frequency and percentage and compared using the chi-square test. A p-value of less than 0.05 was considered statistically significant.

Variable	Category	Seven days	Until delivery	Chi-square value	p-Value
Baby at mother’s side	1-3 days	40 (72.7%)	48 (87.2%)	5.9272	0.2046
	3-5 days	3 (5.4%)	2 (3.7%)		
	5-8 days	2 (3.6%)	2 (3.7%)		
	No	6 (11.0%)	3 (5.4%)		
	Died	4 (7.3%)	0 (0%)		
Duration of stay	1-3 days	29 (52.7%)	17 (30.9%)	9.8193	0.0202
	4-5 days	9 (16.3%)	21 (38.2%)		
	5-10 days	11 (20.0%)	7 (12.7%)		
	>10 days	6 (11.0%)	10 (18.2%)		

## Discussion

This study evaluated the impact of two antibiotic regimens - seven-day therapy versus therapy until delivery - on maternal and neonatal outcomes in women with preterm premature rupture of membranes (PPROM) between 26 weeks 0 days and 36 weeks 6 days of gestation. A total of 110 pregnant women were enrolled at Shri BM Patil Medical College Hospital and Research Centre, Bijapur, India, and randomly allocated into two equal groups. The first group received intravenous ceftriaxone and metronidazole for two days, followed by oral clarithromycin for five days. The second group received the same intravenous regimen, followed by clarithromycin until delivery.

Our findings align with those of Chen et al., who explored different antibiotic protocols in PPROM [11], and extend upon earlier work by Gasparović et al., which demonstrated the benefits of administering antibiotics versus no treatment but did not elaborate on optimal duration [12]. In our cohort, the mean gestational age (GA) at admission was slightly higher in the until-delivery group (33.8 weeks) than in the seven-day group (32.6 weeks), indicating a longer latency period in the extended regimen group. These findings resonate with Gasparović et al., who reported a mean GA of 31.2 weeks in their seven-day antibiotic group [12]. In contrast, Herzlich et al. observed a broader range of gestational ages (17-33 weeks), with a lower mean of 27.1±4.2 weeks, highlighting population variability in PPROM presentations [13].

The need for neonatal respiratory support was comparable between groups in our study. CPAP was required for one to five days in both groups, with only a small fraction of neonates in the until-delivery group requiring support beyond five days (3.6%). Taha et al. reported that 50.6% of neonates required CPAP, and 56.8% used HFNC, supporting the observed need for non-invasive ventilation in neonates with PPROM-associated complications [14]. Ho et al. compared seven- and 14-day regimens but lacked detailed data on respiratory outcomes, limiting comparison [15].

Amniotic fluid levels (AFI) also played a role in neonatal outcomes. In our analysis, 7.2% of participants in the seven-day group had an AFI <5 cm, compared to 14.5% in the until-delivery group. In the seven-day group (n=4), two neonates required CPAP support for respiratory distress, and one neonate required high-flow nasal cannula (HFNC) support. All four neonates survived, and their hospital stay ranged from four to 10 days. In the until-delivery group (n=8), four neonates required CPAP support, one required HFNC, and three required oxygen hood support. No neonatal deaths were recorded in this group, and the hospital stay for these neonates ranged from four to 12 days. These findings are in line with those of Weissmann-Brenner et al., who reported significant differences in outcomes based on AFI <10 cm versus ≥10 cm [16]. Similarly, Vermillion et al. found a significantly shorter latency to delivery in patients with oligohydramnios (AFI <5 cm), suggesting that lower AFI correlates with a higher risk of preterm delivery [17].

NICU admission rates were 47.2% and 41.8% in the seven-day and until-delivery groups, respectively. While these rates were somewhat lower than those reported by Gasparović et al. (68.4%), they are higher than Mercer's control group, which had a 15.6% incidence of neonatal sepsis [12,18]. Importantly, no maternal complications were observed in either group, reinforcing the safety of both antibiotic regimens during the latency period of PPROM.

A key strength of this study is its prospective design with clearly defined antibiotic regimens and uniform inclusion criteria, allowing for a direct comparison of clinical outcomes between two well-matched groups. The study provides valuable insight into the effect of antibiotic duration on latency, delivery mode, and neonatal morbidity in PPROM cases. However, limitations include the modest sample size and the single-center nature of the research, which may limit generalizability. Additionally, the absence of long-term neonatal follow-up precludes conclusions about extended developmental outcomes.

## Conclusions

The study findings show that continuing antibiotics during pregnancy through delivery for PPROM leads to adverse birth complications that increase the need for neonatal respiratory assistance and NICU hospital admission. The administration of antibiotics for seven days does not negatively impact newborn health, yet reduces inappropriate antibiotic treatments. Standardizing antibiotic treatment time in PPROM cases would reduce the risk of antibiotic resistance while protecting neonates from complications, according to these research results.
